# Comparative evaluation of three methods of adhesive remnant removal after orthodontic bracket debonding

**DOI:** 10.1590/2177-6709.27.6.e2220352.oar

**Published:** 2023-03-27

**Authors:** Soghra YASSAEI, Neda JOSHAN, Shiva ABDOLAHY, Azadeh Hakimi Rokn ABADI

**Affiliations:** 1 Sahid Sadoughi University of Medical Sciences, School of Dentistry, Department of Orthodontics (Yazd, Iran).

**Keywords:** Spectrophotometry, Dental debonding, Orthodontic adhesive, Orthodontic bracket

## Abstract

**Objective:**

This study aimed to assess the effects of three methods of adhesive remnant removal (carbide bur and low speed handpiece, carbide bur and high speed handpiece, and zircon-rich glass fiber reinforced composite bur), after orthodontic bracket debonding, on tooth color and enamel surface roughness.

**Methods:**

Ninety sound premolar teeth were selected. The baseline tooth color was assessed using Vita spectrophotometer. The teeth were subjected to bracket bonding processes and then randomly divided into three equal groups. In each group, composite remnant was removed by one of the three methods of adhesive removal, and the teeth were then subjected to color assessment again. To measure the surface roughness, a scanning electron microscope (SEM) with x400 magnification was used.

**Results:**

ANOVA showed that the effect of the three methods of adhesive remnant removal on ∆L, ∆b and ∆E was statistically significant (*p*=0.01), but without significant effect on ∆a. Comparison of the means showed that composite bur and high speed carbide bur yielded the highest ∆E (*p*=0.05), and had a significant difference when compared to carbide bur and low speed handpiece. The highest ∆L and ∆b values belonged to samples approached with composite bur and carbide bur with high speed handpiece, respectively. SEM analysis showed that the composite bur created a very smooth surface, compared to the other two methods.

**Conclusion:**

Zircon-rich glass fiber reinforced composite created the smoothest enamel surface and highest color change, when compared to the other two methods.

## RESUMO

**Objetivo:**

O presente estudo teve como objetivo avaliar os efeitos de três métodos de remoção de adesivo remanescente (broca carbide e peça de mão em baixa rotação, broca carbide e peça de mão em alta rotação, e broca de compósito reforçado com fibra de vidro rica em zircônia), após a descolagem de braquetes ortodônticos, sobre a cor dos dentes e rugosidade superficial do esmalte.

**Métodos:**

Noventa pré-molares hígidos foram selecionados. A cor inicial dos dentes foi avaliada usando um espectrofotômetro Vita. Os dentes foram submetidos à etapa de colagem dos braquetes e, então, divididos aleatoriamente em três grupos. Em cada grupo, o adesivo remanescente foi removido usando um dos três métodos de remoção, e os dentes foram novamente submetidos à avaliação de cor. Para medir a rugosidade superficial dos dentes, foi usado um microscópio eletrônico de varredura (MEV) com aumento de 400x.

**Resultados:**

A ANOVA mostrou que os três métodos de remoção do adesivo remanescente tiveram efeito estatisticamente significativo (*p*=0,01) em ∆L, ∆b e ∆E, mas sem efeito significativo em ∆a. A comparação das médias mostrou que a broca de compósito e a broca carbide em alta rotação produziram o maior ∆E (*p*=0,05) e tiveram uma diferença significativa quando comparadas com a broca carbide e a peça de mão em baixa rotação. Os maiores valores de ∆L e ∆b foram encontrados, respectivamente, nos grupos com broca de compósito e broca carbide usando peça de mão em alta rotação. A análise MEV mostrou que o uso da broca de compósito resultou em uma superfície muito lisa, em comparação com os outros dois métodos.

**Conclusão:**

Em comparação aos outros métodos, a broca de compósito reforçado com fibra de vidro rica em zircônia criou a superfície de esmalte mais lisa e resultou em uma maior mudança de cor.

## INTRODUCTION

The final step in fixed orthodontic treatment is brackets removal from the tooth surface. In this process, the tooth surface is highly vulnerable to irreversible trauma.^1^ Damage to the superficial enamel is concerning for orthodontists, because it comprises the hardest layer and contains the highest amounts of minerals and fluoride. Enamel loss and subsequent exposure of enamel prisms to the oral cavity decreases the enamel resistance to organic acids present in dental plaque, therefore increasing the risk of demineralization. Increased enamel surface roughness and the subsequently increased vulnerability to demineralization, as well as possibility of color change are among the assumed side effects of bracket removal.^2^

Residual adhesive and composite resin remnants after orthodontic bracket debonding can lead to bacterial plaque accumulation, development of periodontal disease, porcelain discoloration, and compromised esthetics.^3^

The adhesive remnants may be removed by different methods, such as tungsten carbide bur and high speed handpiece,^4^ tungsten carbide bur and contra-angle handpiece with high or low speed,^4^ carbide bur and soflex discs with high speed and low speed handpieces^5^ and air abrasion with aluminum oxide.^6^ Currently, use of rotary instruments is the method of choice for adhesive removal.^6^ ^,^ ^7^ Evidence shows that the conventional methods of adhesive removal may cause visible roughness on the enamel surface, create deep gouges with 10-20 µm depth, and result in loss of over 100 µm of underlying enamel.^8^ Aside from the conventional methods, composite burs reinforced with fiber have been suggested for removal of adhesive remnants.^2^ ^,^ ^9^ ^,^ ^10^

Previous studies confirmed that rotational speed is an important factor, because when the tungsten carbide bur was mounted on a low-speed handpiece, less damage was produced than with the high-speed handpiece, which showed the worst performance.^7^ ^,^ ^11^

More recently, ultraviolet light (UV) fluorescent chemicals have been added to orthodontic adhesives to be used as an aid to adhesive remnant removal, but the use of this method with multiblade tungsten-carbide burs did not cause less damage than did conventional lighting.^12^

Color of an object is determined by the light reflection from its surface, and rough surface can cause scattering of the reflected light rays.^13^

The commission international de l’Eclairage (CIE) provided a comprehensive and accurate definition for color as visual perception determined by hue, value and chroma attributes.^14^ Hue is the first aspect of color, related to wavelength of light. This characteristic enables differentiation of colors. Value is probably the most important aspect of color in dental field and determines the whiteness or blackness of the color. Chroma determines the saturation rate of the color.^15^ Currently, objective assessment is a common method for determination of tooth color performed by using digital devices, colorimeters, spectrophotometers and digital image analysis techniques.^13^ ^,^ ^16^ Objective methods often describe the results in CIE Lab system.^14^ The Munsell system is an objective method for assessment of tooth color. In this system, the L* parameter indicates the degree of lightness and ranges from zero (black) to 100 (white). The a* parameter indicates redness or greenness (+a = red, -a = green), and the b* parameter indicates yellowness or blueness (+b = yellow, -b = blue).

Considering the fact that different methods of adhesive removal are associated with some degrees of trauma to the enamel, and no consensus has been reached on an efficient protocol for complete removal of adhesive remnants with minimal trauma and discoloration,^2^ ^,^ ^11^ this study aimed to assess the surface roughness and discoloration of enamel after adhesive removal by three methods: 1) tungsten carbide bur with high speed handpiece; 2) zircon-rich glass fiber reinforced composite bur, and 3) 12-fluted tungsten carbide bur with low speed handpiece. 

## MATERIAL AND METHODS

### SAMPLE PREPARATION

According to Boncuk et al.^9^, which mentioned the standard deviation of ΔE in the two groups of S₁=3.28 and S₂= 2.05, and with a 95% confidence interval (α=0.05) and 80% power (β=0.2), a sample size of 30 in each group was obtained. If any unavoidable failure occurred, another specimen was replaced per group.

A total of ninety sound premolar teeth extracted for orthodontic reasons were collected. The use of the teeth was approved by the ethics and research project committee of the Shahid Sadoughi University of Medical Sciences. Teeth were collected through donations, with consent from the patients (REC number: IR.SSU.REC.1395.179). Then, teeth were then randomly divided into three equal groups, and stored in distilled water.^2^ Maximum duration of storage was one month, and the distilled water was refreshed weekly.^6^ The buccal surface of the teeth was cleaned with non-fluoridated pumice paste and low speed handpiece, rinsed with water and dried with air spray. 

The buccal surface of the teeth was divided into nine segments by drawing lines, and the middle segment was chosen as the enamel window. A standard bracket was bonded to this area, and color assessments were made at this region. To minimize the effect of indirect lights, all assessments were made in the same room under fluorescent light with 1200-1500 lux intensity.^16^

### COLOR ASSESSMENT

The teeth were subjected to color assessment using Vita spectrophotometry (Vita Easyshade^®^ Advance 4.0, Germany). The head of the device was perpendicular to the buccal surface of teeth and in contact with the enamel in the window area. Color measurements were made in triplicate for each tooth, and the mean value was calculated as the color parameter for the respective tooth. The L*, a* and b* parameters were determined for each tooth using Vita spectrophotometer. In this system, the L* parameter indicates the degree of lightness and ranges from zero (black) to 100 (white). The a* parameter indicates redness or greenness (+a = red, -a = green), and the b* parameter indicates yellowness or blueness (+b = yellow, -b = blue).^14^

### BRACKET BONDING, DEBONDING AND RESIN REMOVAL

After primary color assessment, the buccal surfaces of the teeth were etched with 37% phosphoric acid for 30 seconds,^17^ rinsed for 15 seconds and dried, to obtain a chalky white appearance.^2^ A thin layer of adhesive (Resilience Ortho technology, Tamp, Florida, USA) was applied on the etched surface by a micro-brush, and standard stainless steel Edgewise premolar brackets (GAC, Central Islip, New York, NY) were bonded to the enamel surface with composite resin (Resilience Ortho technology, Tamp, Florida, USA) using mild finger pressure. To standardize the bonded area, excess resin and composite around the bracket base were removed, and light-curing was performed from the mesial and distal surfaces for 10 seconds using a LED light-curing unit (Top Light,Taiwan) for a total of 20 seconds, using 5W of power output and effective area of 4mm depth. The samples were immersed in water at 37°C for 24 hours.^18^ Brackets were then debonded using bracket removal pliers (Ormco, Glendora, CA, USA). The teeth were then randomly divided into three equal groups. In group 1, adhesive remnants were removed using the conventional method; i.e. low speed handpiece (M101,W&H Adec, Australia-max, up to 40,000 rpm) and 12-fluted tungsten carbide bur (Geber. Brasseler, Komet-Lemgo, Germany). In group 2, tungsten carbide bur (Geber. Brasseler, Komet-Lemgo, Germany) with high speed handpiece (M101,W&H Adec, Australia, 200,000 rpm) was used for adhesive remnant removal. In group 3, composite bur reinforced by zircon-rich glass fiber (Stainbuster, Abrasive Technology Inc., Lewis Center, Ohio, USA) was used with low speed handpiece to remove adhesive remnants from the tooth surface. After adhesive removal, the teeth were subjected to color assessment again.

### ASSESSMENT OF COLOR CHANGE

The CIE Lab system was used for interpretation of color change of teeth. To obtain color change (ΔE), the following formula was used:

$$\Delta E\;=\;{\left[{\left(\Delta L\right)}^{2}+\;{\left(\Delta a\right)}^{2}+\;{\left(\Delta b\right)}^{2}\right]}^{0.5}$$

Where ΔL indicates change in lightness, Δa indicates changes in greenness-redness, and Δb indicates changes in yellowness-blueness. In this study, the clinically perceptible threshold of color change (ΔE) was considered to be 3.7.^19^

### ASSESSMENT OF SURFACE ROUGHNESS

The teeth were evaluated under a scanning electron microscope (SEM) at x200 and x400 magnifications, to determine enamel surface roughness. The enamel surface was gold coated and examined in a scanning electron microscope with an accelerating voltage of 20 kV. The surface changes were interpreted from adapted enamel damage index described by Howell and Weekes.^20^ In our index, grade A refers to smooth enamel surface with no scratches, grade B refers to an acceptable surface with a few scattered scratches, grade C refers to a rough surface with several rough and deep scratches or a few visible scratches, and grade D refers to coarse deep and wide scratches, with enamel damage visible to the naked eye. 

### STATISTICAL ANALYSIS

Data were analyzed using SPSS v.17. Statistical comparisons were made using ANOVA, and Scheffe test was used for pairwise comparisons (*p*<0.05 was considered statistically significant). The results were analyzed with 95% confidence interval. 

## RESULTS

### RESULTS OF COLOR ASSESSMENTS

ANOVA showed that the effect of the three methods of adhesive removal on ΔL parameter was statistically significant (*p*=0.003). The highest ΔL was noted for composite bur (ΔL=19.87), and the lowest for high speed tungsten carbide bur (ΔL=-5.57) (Table 1).

**Table 1:** Assessment of color parameters in the three groups.

Parameter	Method	Number	Minimum	Maximum	Mean ± SD	P-value	Significance
∆L	Carbide, low speed	30	-8.43	6.03	0.79 ± 4.10	0.003	**
	Carbide, high speed	30	-5.57	17.70	1.03 ± 5.13		
	Composite bur reinforced with zircon-rich glass fiber	30	-6.57	19.87	5.31 ± 7.19		
	Total	90	-8.43	19.87	2.38 ±5.94		
∆a	Carbide, low speed	30	-10.93	0.97	-3.39 ± 3.01	0.089	NS
	Carbide, high speed	30	-12.17	-1.30	-4.62 ± 2.35		
	Composite bur reinforced with zircon-rich glass fiber	30	-8.80	2.57	-2.9 ± 3.74		
	Total	90	-12.17	2.57	-3.64 ± 3.14		
∆b	Carbide, low speed	30	-12.93	2.83	-2.16 ± 3.14	0.002	**
	Carbide, high speed	30	-15.37	0.13	-6.81 ± 4.24		
	Composite bur reinforced with zircon-rich glass fiber	30	-20.00	7.77	-5.04 ± 6.73		
	Total	90	-20.00	7.77	-4.67 ± 5.25		
∆E	Carbide, low speed	30	0.82	14.31	6.38 ± 3.31	0.002	**
	Carbide, high speed	30	3.08	26.41	9.86 ± 4.53		
	Composite bur reinforced with zircon-rich glass fiber	30	1.03	23.30	11.12 ± 6.87		
	Total	90	0.82	26.41	9.12 ± 5.45		

One-way ANOVA, SD = standard deviation; NS = not significant. *p < 0.05; **p < 0.01. (statistically significant for p<0.05).

**Figure 1:**
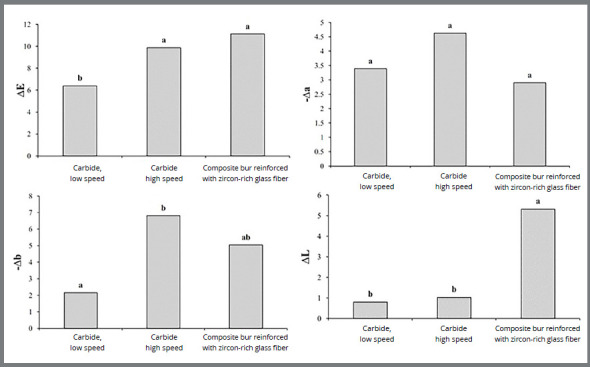
Comparison of the mean effect of the three methods on color parameters using Scheffe test at 5% level of significance.

Pairwise comparisons by the Scheffe test showed that ΔL in groups 1 and 2 was not significantly different, but group 3 had significantly higher ΔL than groups 1 and 2 (Fig 1).

Regarding Δa, ANOVA and Scheffe tests showed no significant difference among the three groups (*p*=0.089, Table 1) (Fig 1).

ANOVA showed a significant difference in Δb among the three methods (*p*=0.002). The highest Δb belonged to composite bur (Δb=-20) and the lowest Δb was noted for tungsten carbide bur (Δb=0.13) (Table 1). Comparison of the mean values using the Scheffe test showed that Δb in group 2 was significantly higher than that in group 1. No significant difference was noted between groups 3 and 2 or between groups 3 and 1 (Fig 1).

The results of ANOVA showed a significant difference among the three methods in ΔE (*p*=0.002, Table 1). The highest ΔE belonged to group 2 (23.3) and the lowest was noted in group 1 (0.82) (Table 1). Pairwise comparisons by the Scheffe test showed significant differences between groups 3 and 1 and also between groups 2 and 1 (Fig 1). 

### RESULTS OF SURFACE ROUGHNESS ASSESSMENT

**Figure 2:**
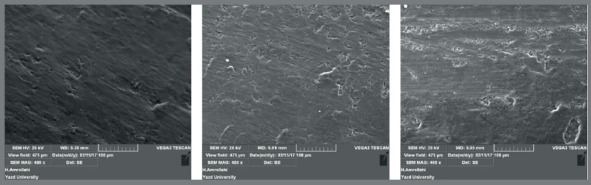
Surface roughness of samples after resin removal by carbide bur and low speed handpiece (x400 magnification).

**Figure 3:**
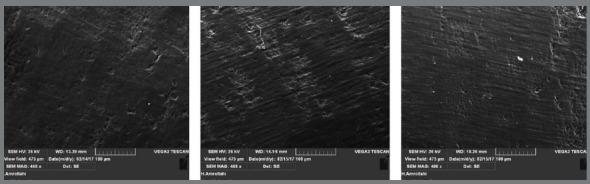
Surface roughness of samples after resin removal by carbide bur and high speed handpiece (x400 magnification).

**Figure 4:**
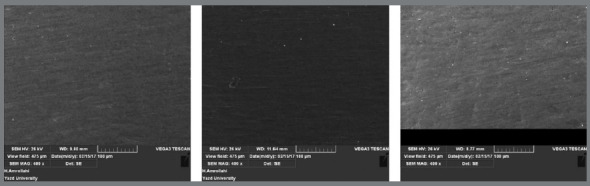
Surface roughness of samples after resin removal by composite bur reinforced with zircon-rich glass fiber (x400 magnification).

SEM micrographs showed that surface roughness was grade B in low speed tungsten carbide bur (Fig 2), grade C in high speed tungsten carbide bur (Fig 3), and grade A in composite bur (Fig 4).

## DISCUSSION

Tooth color of the anterior teeth is an important parameter in smile esthetics.^21^ Enamel color alterations after orthodontic treatment may be related to the post-debonding resin removal.^22^ Several factors, e.g. type of adhesive resins, incorrect debonding instruments and techniques, may be responsible for enamel damage.^5^

Studies on different methods of adhesive remnant removal are limited, and the available ones have reported controversial results. Some authors reported that bracket bonding and debonding processes have no significant effect on the human enamel,^23^ while some recent studies reported enamel discoloration as the result of procedures performed during orthodontic treatment.^1^ ^,^ ^22^ Boncuk et al.^9^ reported that both bonding and adhesive remnant removal can change the tooth color. Several studies have assessed different techniques of bracket debonding, resin removal and enamel surface polishing.^10^ The present study aimed to evaluate tooth discoloration after adhesive removal by three different methods: 1) carbide bur with high speed handpiece, 2) carbide bur with low speed handpiece and 3) composite bur reinforced with zircon-rich glass fiber. The first two methods are commonly used for adhesive remnant removal, but the third method is still under investigation.

Easy shade compact spectrophotometer (VITA Zahnfabrik) was used for accurate and reliable measurement of color in the current study. To increase the accuracy of color assessment, three measurements were made for each tooth and the mean L*, a* and b* values were calculated. 

The present results showed that the three methods increased ΔL and this parameter was 0.79, 1.03 and 5.31 for the three methods of resin removal, which indicates that ΔL after the use of the third method was significantly higher than that in the other two groups.

The surface roughness is connected directly with the brightness and, thus, the esthetics of the teeth.^1^ In the present study, SEM results showed that the third method yielded a much smoother surface, with less roughness. Surface roughness determines mirror reflection of light and causes lightness,^24^ this finding resulted in an increase in whiteness in group 3. Also, since ΔL<2 is not clinically perceivable^25^ and the mean ΔL in group three was higher than 2, the lightness of teeth in group 3 was significantly higher. This finding is in agreement with that of Cörekçi et al.^26^ study. However, it should be noted that they assessed the effect of type of adhesive on tooth color. SEM assessment of enamel surface only shows surface topography, which is not quantitative and cannot be used for the purpose of comparison. However, SEM assessments enable visual comparison of the efficacy of resin remnant removal methods.^2^

The Δa parameter, which indicates greenness-redness, was not significantly different among the three methods in the present study, but it should be noted that all three methods decreased Δa (made it negative) and thus, shifted the color towards green. Also, Δb, which indicates yellowness-blueness, became negative in all three methods, and indicated a shift towards blue. 

Cörekçi et al.^26^ reported that the use of different composite resins for bracket bonding increased the mean a* and decreased the mean b* parameter. Kim et al.^27^ evaluated the effect of bracket bonding and debonding on the enamel color, and reported that ΔL decreased by 4.58 units, while Δa and Δb increased by 0.32 and 2.06 units, respectively.

Different studies have reported different values for clinically perceptible threshold of color change (ΔE) and ΔE=2.72,^28^ ΔE=3.7^29^ and ΔE=3.46^30^ have been reported as the clinically perceptible threshold. In the current study, ΔE=3.7 was considered as the clinically perceptible threshold of color change. The results showed that ΔE in all three methods was over 3.7, which indicates enamel color change in all three methods. ΔE in the groups 2 and 3 was not significantly different, but both methods caused greater color change than the first method. Considering ΔL and Δb values, the third method yielded the highest ΔL, while the second method yielded the highest Δb; however, the first method yielded the lowest ΔL and Δb. Thus, color change in group 3 was due to a shift towards lightness, but color change in group 2 was due to color shift towards the blue. Eliades et al.^22^ and Kim et al.^27^ reported that the bonding and resin removal methods increased ΔE by 5.27 to 13.7 units, which was in agreement with the present results. Wriedt et al.^31^ and Trakyali et al.^19^ believed that bonding and debonding processes do not cause human enamel discoloration.

Regarding the results of the present study, composite bur with air or water cooling system is a worthy technique to remove the residual adhesive after orthodontic bracket debonding. This result is in line with those obtained by Erdur et al.^32^ and Arbutina et al.,^33^ who reported that a composite bur delivered a smoother enamel surface, in comparison to a tungsten carbide bur. Also, Cardoso et al.^34^ reported that a composite bur and a Sof-Lex disc associated with polishing are recommended, due to little damage to the enamel. 

According to the present study, composite bur increases the lightness parameter, and thereby increases the color change, which means that the teeth are whiter due to the creation of a smooth enamel surface that affects the reflection of light from the tooth surface. This advantage can reduce the need for treatments such as bleaching at the end of orthodontic treatment. However, it is still difficult to extrapolate the results directly from the *in-vitro* to the *in-vivo* situations. These results provide clinically useful information, but we emphasize that more clinical studies are needed to confirm the results.

## CONCLUSION

1. SEM images showed that composite bur created the smoothest enamel surface, while low speed handpiece and tungsten carbide bur created the roughest.
2. Color assessment revealed that lowest and highest color change were observed in low speed handpiece, carbide bur and zircon-rich glass fiber reinforced composite bur, respectively.
